# Synaptic Impairment and Robustness of Excitatory Neuronal Networks with Different Topologies

**DOI:** 10.3389/fncir.2017.00038

**Published:** 2017-06-13

**Authors:** Ehsan Mirzakhalili, Eleni Gourgou, Victoria Booth, Bogdan Epureanu

**Affiliations:** ^1^Department of Mechanical Engineering, University of MichiganAnn Arbor, MI, United States; ^2^Division of Geriatrics, Department of Internal Medicine, Medical School, University of MichiganAnn Arbor, MI, United States; ^3^Department of Mathematics, University of MichiganAnn Arbor, MI, United States; ^4^Department of Anesthesiology, Medical School, University of MichiganAnn Arbor, MI, United States

**Keywords:** persistent activity, synaptic impairment, robustness, rich club, small world network

## Abstract

Synaptic deficiencies are a known hallmark of neurodegenerative diseases, but the diagnosis of impaired synapses on the cellular level is not an easy task. Nonetheless, changes in the system-level dynamics of neuronal networks with damaged synapses can be detected using techniques that do not require high spatial resolution. This paper investigates how the structure/topology of neuronal networks influences their dynamics when they suffer from synaptic loss. We study different neuronal network structures/topologies by specifying their degree distributions. The modes of the degree distribution can be used to construct networks that consist of rich clubs and resemble small world networks, as well. We define two dynamical metrics to compare the activity of networks with different structures: persistent activity (namely, the self-sustained activity of the network upon removal of the initial stimulus) and quality of activity (namely, percentage of neurons that participate in the persistent activity of the network). Our results show that synaptic loss affects the persistent activity of networks with bimodal degree distributions less than it affects random networks. The robustness of neuronal networks enhances when the distance between the modes of the degree distribution increases, suggesting that the rich clubs of networks with distinct modes keep the whole network active. In addition, a tradeoff is observed between the quality of activity and the persistent activity. For a range of distributions, both of these dynamical metrics are considerably high for networks with bimodal degree distribution compared to random networks. We also propose three different scenarios of synaptic impairment, which may correspond to different pathological or biological conditions. Regardless of the network structure/topology, results demonstrate that synaptic loss has more severe effects on the activity of the network when impairments are correlated with the activity of the neurons.

## Introduction

The network structure/topology of the brain plays an indisputable role in a wide variety of tasks the brain performs (Sporns, 2010). Knowledge of the function of brain networks enables the development of tools and methods to detect and treat pathological conditions related to network malfunction (Morgan and Soltesz, 2008; Crossley et al., 2014). That is in part why building the human connectome has attracted vast efforts in the past few years (Van Essen et al., 2013; Hodge et al., 2016).

Although the complete human connectome is not available yet, even mapping individual circuits of humans or other animals central nervous system has provided researchers with enormous amount of data to study the network structure/topology of the brain. The non-random structure of the brain networks is a common conclusion of all these studies (Sporns, 2010). Scale-free or small-world network structures of the brain have been proposed (Eguíluz et al., 2005; Achard, 2006; He et al., 2007; van den Heuvel et al., 2008). More recently, the neuronal network of *C. elegans*, the first organism to have its connectome fully mapped (White et al., 1986; Varshney et al., 2011), has revealed the presence of hubs and rich clubs in its nervous system (Towlson et al., 2013). Although in less detail, mesoscale and macroscale studies in humans (van den Heuvel and Sporns, 2011), mice (Oh et al., 2014) and cats (de Reus and van den Heuvel, 2013) have shown similar findings. Additionally, such heterogeneity in the network structure has been observed also in cultured cells, and emergence of small-world networks (Downes et al., 2012) or rich clubs (Schroeter et al., 2015) has been reported for *in vitro* experiments.

The number and the strength of connections that neurons make with each other, create a non-random spatial topology of neuronal networks (Fornito et al., 2013). Connections are not completely static even in normal conditions. For example, synaptic plasticity and neuromodulation can affect the dynamics of neuronal networks and change the brain's functional connectivity (Abbott and Nelson, 2000; Marder and Thirumalai, 2002). Malfunctions in synaptic connections and synaptic dynamics can jeopardize the normal functionality of brain. For instance, studies have indicated loss of synapses as a hallmark of Alzheimer's disease (Selkoe, 2002; Shankar and Walsh, 2009; Sheng et al., 2012). Random loss of synapses has already been used to simulate different stages of Alzheimer's disease (Abuhassan et al., 2014). However, there are biological or pathological conditions that can lead to non-random loss of synapses. For instance, recent studies have shown that oxidative stress related to neuronal activity may result in dysfunction of synapses in Alzheimer's disease (Kamat et al., 2016).

EEG (Gaál et al., 2010; Smit et al., 2010) and fMRI (Wang et al., 2010; Zhu et al., 2012; Wu et al., 2013) data have revealed that the network topology of the brain changes even during normal aging. Therefore, the study of neurodegenerative diseases and their indications on the network level can be the key to better understanding of these disorders by monitoring the structure and dynamics of neuronal networks (Palop et al., 2006; Kosik, 2013; Kocher et al., 2015). In the present study, we investigate the impact of synaptic deficiency, namely reduced effectiveness of the synaptic function, on the robustness of neuronal networks with different topologies. The biological process that leads to this deficiency can be attributed to many factors, ranging from functional decline during normal aging, to age-associated neurodegenerative diseases, such as Alzheimer's disease. We focus on networks with only excitatory neurons to emphasize on the importance of topology on the robustness of networks, since inhibitory neurons are shown to increase networks' robustness (Petersen et al., 2014). Particularly, we focus on persistent activity of neuronal networks defined as the ability of the network to sustain its activity upon removal of the initial stimulation. Persistent activity has been linked to working memory, which is the ability to remember information for periods of time in the order of seconds (Baddeley, 1992; Sakai et al., 2002; Curtis and D'Esposito, 2003). One of the reasons why we are interested in persistent activity is because working memory is known to be adversely affected in patients with Alzheimer's disease (Baddeley et al., 1991, 2001; Stopford et al., 2012). Previously, network connectivity (Roxin et al., 2004; Shanahan, 2008) and degree distribution of networks (Roxin, 2011) has been shown to influence the self-sustained activity of neuronal networks under normal conditions. However, there are no studies where the sensitivity of persistent activity to synaptic failure is investigated. We show here that networks with rich clubs can be constructed systematically by using bimodal degree distributions. Then, we examine how networks with different topologies respond to different levels and types of impairment. Robustness and capability of the neuronal network to maintain activity are used to differentiate dynamics of networks with different topologies.

## Methods

### Neuron model

We adopt a neuron model based on the Hodgkin-Huxley formalism. The model features a fast Na^+^ current, a delayed rectifier K^+^ current and a leakage current (Amitai, 1994; Stiefel et al., 2009; Rich et al., 2016). The current balance equation for cell *i* is:

(1) $$\begin{array}{r} C\frac{dV_{i}}{dt}=-g_{Na}m_{\infty }^{3}h\left(V_{i}-E_{Na}\right)-g_{Kdr}n^{4}\left(V_{i}-E_{K}\right)\\ -g_{L}\left(V_{i}-E_{L}\right)+I_{ext}-I_{i}^{syn}, \end{array}$$

where *C* = 1.0 μF/cm^2^, *g*_*Na*_ = 24.0 mS/cm^2^, $g_{Kdr}=3.0\text{ mS}/\text{c}{\text{m}}^{2}$, $g_{L}=0.02\text{ mS}/\text{c}{\text{m}}^{2}$, *E*_*Na*_ = 55.0 *mV*, *E*_*K*_ = −90.0 mV, *E*_*L*_ = −60.0 mV (Amitai, 1994; Stiefel et al., 2009; Fink et al., 2011). *I*_*ext*_ is the external current (measured in μA/cm^2^) that controls the firing frequency of the neuron. This current is chosen so that the firing frequency of the *ith* cell is zero when $I_{i}^{syn}$ (the synaptic current received by neuron *i*) is zero.

The Na^+^ inactivation gating variable *h*, and the K^+^ delayed rectifier activation gating variable *n* are governed by first order dynamic equations expressed as:

(2) $$\begin{array}{r} \frac{dh}{dt}=\frac{h_{\infty }-h}{\tau_{h}}\text{and }\frac{dn}{dt}=\frac{n_{\infty }-n}{\tau_{n}}, \end{array}$$

where the steady state values and the time constants are given by:

(3) $$\begin{array}{r} h_{\infty }=\frac{1}{1+\exp\left(\frac{V+53.0}{7.0}\right)}, \end{array}$$

(4) $$\begin{array}{r} \tau_{h}=0.37+\frac{2.78}{1+\exp\left(\frac{V+40.5}{6.0}\right)}, \end{array}$$

(5) $$\begin{array}{r} n_{\infty }=\frac{1}{1+\exp\left(\frac{-\text{V}-30}{10.0}\right)}, \end{array}$$

(6) $$\begin{array}{r} \tau_{n}=0.37+\frac{1.85}{1+\exp\left(\frac{V+27.0}{15.0}\right)}. \end{array}$$

The activation of Na^+^ current is assumed instantaneous, and is modeled by the following function:

(7) $$\begin{array}{r} m_{\infty }=\frac{1}{1+\exp\left(\frac{-V-30}{9.5}\right)}. \end{array}$$

### Synaptic model

The synaptic current from the presynaptic neuron *j* received by the postsynaptic neuron *i* is modeled as:

(8) $$\begin{array}{r} I_{ij}^{syn}=g_{syn}W_{ij}s_{ij}\left(V_{i}-E_{s}\right), \end{array}$$

where $g_{syn}=0.005\text{ mS}/\text{c}{\text{m}}^{2}$ is the maximum synaptic conductance, and *E*_*s*_ is the reversal potential for the synaptic current, which is usually considered equal to zero for excitatory synapses. *W*_*ij*_ is the connectivity weight between the presynaptic neuron *j* and the postsynaptic neuron *i*. A synaptic connection without any synaptic impairment has a weight of *W*_*ij*_ = 1. When *W*_*ij*_ = 0, the two neurons are not connected to each other. We define synaptic deficiencies as any values of *W*_*ij*_ between 0 and 1. *s*_*ij*_ is the fraction of open receptors, which follows a simple first order kinetic equation given by:

(9) $$\begin{array}{r} \frac{ds_{ij}}{dt}=\alpha \left[T_{j}\right]\left(1-s_{ij}\right)-\beta s_{ij}, \end{array}$$

Here α = 1.1 mM^−1^ms^−1^ and β = 0.19 ms^−1^ (Destexhe et al., 1998) are constants and correspond to forward and backward rates. [*T*_*j*_] is the concentration of neurotransmitters released by the presynaptic neuron *j* that can be approximated by the following equation (Destexhe et al., 1998):

(10) $$\begin{array}{r} \left[T_{j}\right]=\frac{T_{max}}{1+\exp\left(-\frac{V_{j}-V_{p}}{K_{p}}\right)}, \end{array}$$

where *T*_*max*_ = 1 mM is the maximum concentration of released neurotransmitters by the presynaptic neuron. *K*_*p*_ = 5 mV and *V*_*p*_ = 2 mV are constants that determine the steepness and half-activation value of the neurotransmitter release (Hass et al., 2016).

The total synaptic current received by neuron *i* is the summation of all synaptic currents from its presynaptic neurons in the network.

### Network connectivity

We use the degree distribution of networks to construct networks with different topological metrics. While small world networks can be constructed systematically (Watts and Strogatz, 1998) without using the degree distribution of the network, authors are not aware of such methods to build networks that consist of rich clubs. Hence, we propose a method in which networks with rich clubs can be constructed, provided that they are based on bimodal degree distributions.

The degree of a neuron is defined as the summation of its indegrees (number of its inputs) and outdegrees (number of its outputs). The indegree and outdegree of a neuron can be different since the connections are not necessarily bidirectional. To generate networks with different degree distributions, we chose to concentrate on bimodal distributions, because they are the simplest distributions that are not single modal. Without any constraints, the degree distribution of a purely random network, also known as an Erdös–Rényi model, follows a Poisson distribution (Erdös and Rényi, 1959). The networks in this study have 200 neurons, with 5% probability of connectivity. The probability of connectivity is defined as the chance of having a unidirectional connection between two neurons in the network. For the above parameters, a totally random network has a single mode distribution of mean 20. To make a close comparison to single modal distributions, we create bimodal distributions that have the same mean. Taking this as a reference case, all other networks are created so as to have the same number of synapses but with different mode distributions. To this end, the following relation for the modes of networks with bimodal degree distribution must hold:

(11) $$\begin{array}{r} \omega_{1}M_{1}\;+\;\omega_{2}M_{2}=20, \end{array}$$

where ω_1_ and ω_2_ are weights of the modes and *M*_1_ and *M*_2_ are the mean degree values of the modes. Hence, if weights of the modes are equal, then their average must be equal to 20 (for example *M*_1_ = 10 and *M*_2_ = 30 are a valid pair when the weights are equal).

**Figure 1:**
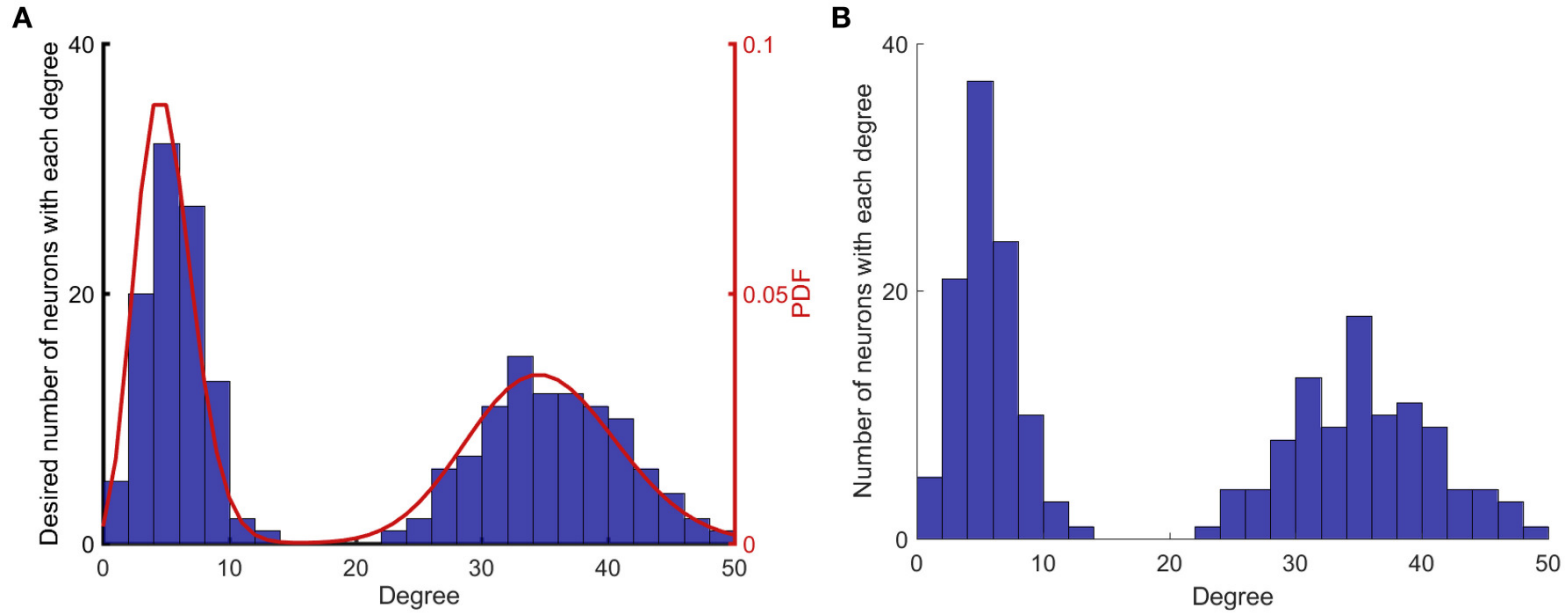
Implementation of network construction with desired degree (summation of indegree and outdegree) distribution. **(A)** A degree distribution that matches the desired probability distribution function (PDF) is randomly generated. **(B)** A network that matches the desired degree distribution described in **(A)**, is randomly generated. The algorithm generates networks that match the desired degree distribution in a non-exact manner, since the process is random.

To generate networks with different degree distributions, first, two Poisson distributions with the desired mean values are generated. Then, each of the probability distributions is normalized and weighted as desired so that the integral of the combined bimodal probability distribution function (PDF) is equal to 1. Next, a bin size is chosen, and the combined PDF is integrated over each bin. The result of the integration in each bin shows the number of neurons that must have degrees between limits of that bin. Next, the proper number of neurons is assigned randomly with degrees according to the limits of each bin. Thus, the PDF is converted to a degree distribution for the anticipated network. The next step is to construct a network according to the established degree distribution. For this purpose, a scrambled list is created in which each neuron is repeated at a number of times equal to its degree (Cohen and Havlin, 2010). Next, two non-identical members of the list are selected randomly, and a connection is created from the first to the second element. Then, these two elements are removed from the list, and the process is continued until the list is empty. With this approach, a directed graph without any self-loops is constructed with the desired degree distribution. Figure 1 shows the implementation of this method to construct a network with mean values of 5 and 35, with equal weights.

Figure 1 shows the steps that are taken to construct networks with different degree distributions. Figure 1A shows the first two steps that are prescribing a PDF and creating a random degree distribution according to that PDF. Figure 1B shows the degree distribution of the actual network that is constructed based on the degree distribution in Figure 1A. The degree distributions shown in Figures 1A,B are different, because the process of generating a network for a given degree distribution is random.

In addition to random networks with a single mode with mean degree value of 20, three bimodal degree distributions with pairs of {15, 25}, {10, 30}, and {5, 35} are used in this study. Even though the two mean values used to build each distribution are imposed, the way that the lists are assembled and then networks are created is random. Therefore, the whole process of network construction is random, which leads to deviations in the number of synapses among different networks. Nevertheless, these deviations are small and negligible as the number of synapses is mainly a function of the network size and the probability of connectivity, which both remain unchanged in this study. However, to minimize the effects of stochasticity arising from the process of network construction on the system's dynamics, 50 realizations of each degree distribution are used to obtain the results.

### Impairment modeling

To quantify synaptic impairment of the network, we define two metrics: the level of impairment and the percentage of impairment. Impairments are implemented in the elements of the adjacency matrix *W*, which represent the synapses in the network. When the element *W*_*ij*_ is zero, then the two neurons *i* and *j* are not connected. When the element *W*_*ij*_ is nonzero, then the postsynaptic neuron *i* receives an input from the presynaptic neuron *j*. If the nonzero element is equal to 1, then the synapse between the two neurons is considered to be healthy, i.e., it has full strength. If the nonzero element is <1, then the synaptic connection is considered impaired, and the difference between the nonzero element and 1 is defined as the level of impairment. The percentage of impairment indicates what percentage of synapses in the network is weakened by the specific level of impairment. For example, a level of impairment of 0.6 and a percentage of impairment of 20% indicate that 20% of nonzero elements of the adjacency matrix of the network have *W*_*ij*_ = 0.4. For the same level and percentage of impairment, three possible scenarios of deficiency are used to study the effects of synaptic deficiency in the network.

In the first impairment scenario, synapses are randomly selected and weakened or removed, with equal probability. Conditions in which all neurons in a network can be affected equally may lead to random weakening of synapses. For example, it is possible that aging affects neurons in some regions of the brain with equal likelihood, and weakens the synapses randomly. Such method of synaptic weakening has been also used to model different stages of Alzheimer's disease (Abuhassan et al., 2014). In addition, random impairments are analogs to normal heterogeneity in the strength of synapses in healthy neuronal networks. Furthermore, random impairments can be considered as the control scenario to determine if other impairment scenarios show different results.

In the second impairment scenario, neurons that have a higher number of synapses are more likely to be weakened or removed. The hypothesis for this type of defect is based on the significance of intracellular transport. We speculate that such impairment can be linked to pathologies where axonal transport is not functioning properly (De Vos et al., 2008). For instance, tau protein has been proposed to cause synapse loss induced by impaired axonal transport (Kopeikina et al., 2013). Axonal transport is required to provide precursor proteins that are essential for production and recycling of synaptic vesicles (Rizzoli, 2014). Therefore, the load and efficiency of axonal transport is related to the number of functional synapses a neuron can maintain. For a neuron with few synapses, impaired axonal transport may still allow for synapses to be functional. However, if the number of synapses of the same neuron increases, the already impaired axonal transport becomes overloaded as well and thus it becomes more difficult for the neuron to perform. Hence, we propose that the synapses of neurons with large number of out-going synapses are more likely to be weakened in case of inefficient axonal transport. To implement this impairment scenario, the outdegree of all neurons in the network is calculated and sorted. Next, neurons with higher outdegree are selected and impaired first.

**Figure 2:**
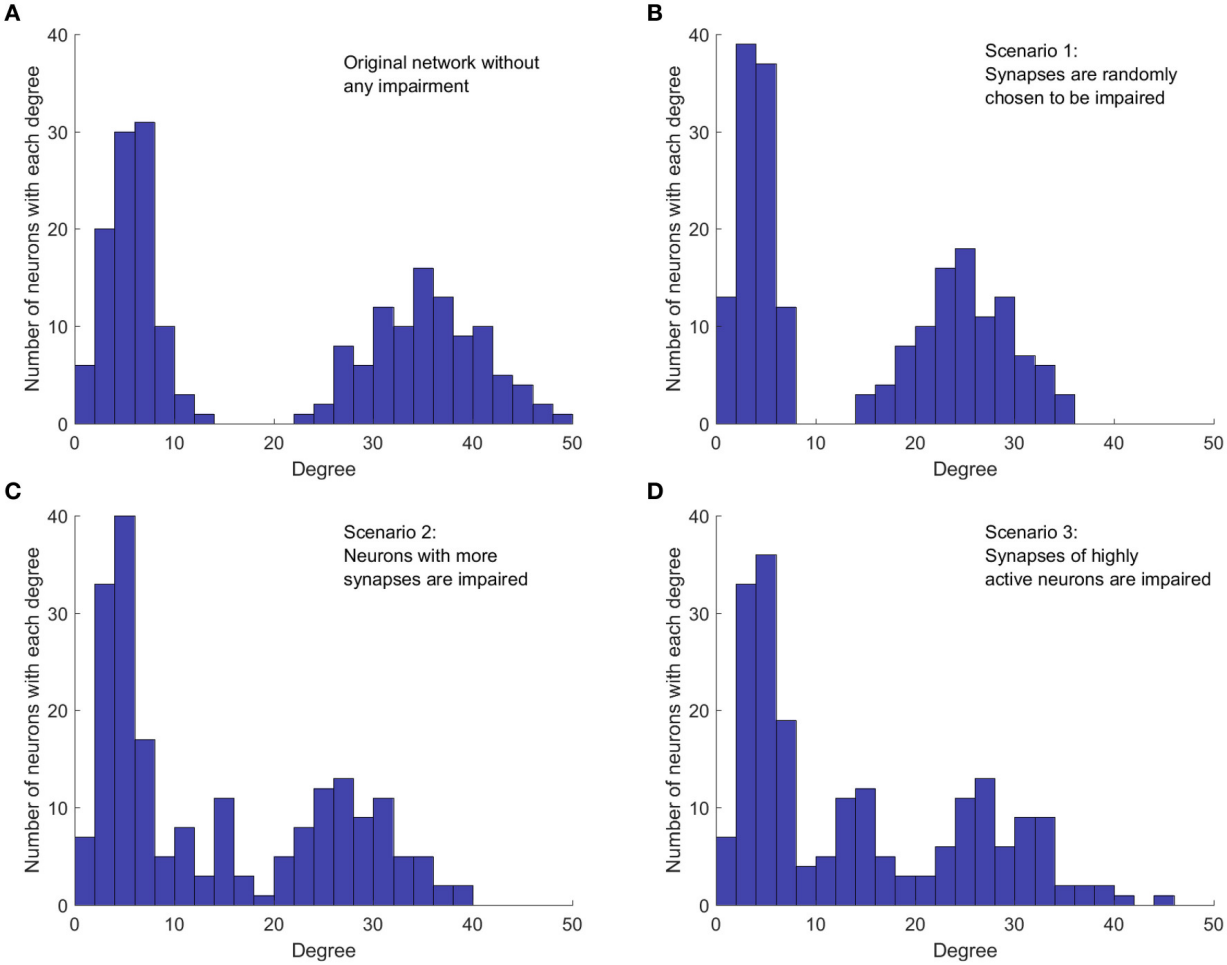
Implementation of the three cases of network impairment. Each graph shows how the original bimodal degree (summation of indegree and outdegree) distribution is affected in different impairment scenarios. Each method of impairment affects the original network degree distribution differently, leading to different network dynamics. Level of impairment and percentage of impairment are 1 and 30%, respectively, for all impairment scenarios. **(A)** Degree distribution in the original network, without any impairment. Network is constructed with mean values 5 and 35, with equal weights. **(B)** Random impairment of synapses. The shape of distribution and the general structure of the network are not affected. **(C)** Synaptic impairment based on the number of synapses per neuron. Neurons with more synapses are affected more. Shape of distribution and overall network structure are also affected. **(D)** Synaptic impairment based on neuron's level of activity. More active neurons suffer more. Distribution and network structure are changed.

In the third impairment scenario, synapses of neurons that are highly active are more likely to be weakened or removed. Thus, this scenario considers the activity of neurons, not just the network topology. This contrasts the second scenario where neurons with more synapses are more likely to suffer from inefficient axonal transport. If such a neuron is not firing frequently, then even an impaired axonal transport might be capable of keeping synapses functional. Nevertheless, if such a neuron is highly active and fires frequently, then defective axonal transport will result in more ineffective synapses compared to a less active neuron which fires less frequently. In fact, synaptic fatigue has already been seen in experimental results even in healthy neurons for high-frequency stimulation (Pozzo-Miller et al., 1999). To this end, the third impairment scenario investigates how impairment of synapses of highly active neurons affects the activity of the whole network. To explore such synaptic weakening, the number of firings for each neuron in the unperturbed network is measured over a fixed period of time. Next, neurons are sorted based on their level of activity and those that are more active are selected first to have their synaptic weights reduced. Figure 2 shows an example of how each scenario affects the degree distribution of a network. The healthy/unperturbed network is constructed using mean values of 5 and 35 with equal weights. The level and percentage of impairments are 1 and 30%, respectively, for all impairment scenarios.

Comparing the degree distribution of the original network (Figure 2A) with the distribution in the first impairment scenario (Figure 2B), we note that, as expected, the random selection affects neither the structure of the network, nor the shape of the degree distribution. However, the mean degree value of each mode and consequently, the mean degree value of the whole network decrease because of the applied impairments. In the second impairment scenario (Figure 2C), the mode with higher mean degree is affected more, since neurons with higher degrees are targeted first. Moreover, removing synapses from neurons with higher outdegree increases the number of neurons with lower degrees. Therefore, the height and width of the first mode increases in this scenario. In the third impairment scenario (Figure 2D), neurons with higher degrees are still more likely to be affected since those with higher degrees are generally more active. However, comparing the degree distribution of the second and the third scenarios, we notice that they are not equivalent. For instance, in the third impairment scenario, neurons with a degree of 45 still exist in the impaired network while such neurons are removed in the second impairment scenario. Importantly, the presence of such neurons in the third impairment scenario shows that neurons with a high degree are not necessarily more active. This is expected, because the activity of a neuron in the network depends on the dynamics of the whole network.

### Topological metrics

If a neuronal network is considered as a graph, each neuron is a node and the synaptic connection between each two neurons is an edge. Then, several metrics can be used to describe features of the network based on graph theory (Rubinov and Sporns, 2010). The degree of a node discussed above is the simplest of these metrics. Another metric is the characteristic path length defined as the average of path lengths (minimum number of edges between two nodes) over the whole network. Another metric is the clustering coefficient defined as the ratio of closed paths of length 2 over the total paths of length 2 in the whole network (Newman, 2010). A network with a characteristic path length comparable to a random network but with a higher clustering coefficient is known to be a small world network (Watts and Strogatz, 1998). Another metric is the rich club coefficient defined over degrees of the network (Zhou and Mondragon, 2004; Colizza et al., 2006; McAuley et al., 2007). For a chosen degree *k*, all of the nodes with smaller degrees than *k* and their corresponding edges are removed from the network. Then, the rich club coefficient is defined as the ratio of the edges in the remaining set over the number of edges in a fully connected network of the same size. The rich club coefficient is not a metric that can be defined for the whole network, since it depends on the chosen degree *k*, which varies between the lowest and highest degrees of nodes in networks. For each *k*, the size of the remaining network (club size) can be used to show how the rich club coefficient varies within a network. The network size and probability of connectivity can affect all these metrics. Thus, the network metrics of a purely random network have been used to normalize these metrics for different network structures/topologies (McAuley et al., 2007).

### Dynamical metrics

Unlike topological metrics, dynamical metrics depend on the activity of the neurons and their intrinsic properties, such as their excitability. Network synchronization is one of the most widely used dynamical metrics that has been used for the investigation of complex networks (Barrat et al., 2008). However, network synchronization is not an informative metric in our case, because the neuron model used in this work is a type 1 neuron and networks that consist of such neurons are shown to have asynchronous behavior (Fink et al., 2011).

The first dynamical metric we use, is the presence of persistent activity. Persistent activity is a collective behavior of a network that indicates whether the network can sustain its activity for long periods of time, once the initial stimulus is removed. To quantify persistent activity, we declare that a network has persistent activity if even a single neuron has fired at least once during a time window (i.e., the last 200 ms) at the end of a longer period (e.g., a period of 4,000 ms).

All levels of impairment and percentages of impairment for each network need to be examined to determine the sensitivity of the persistent activity to impairments. However, this approach is computationally inefficient. A more effective approach is to estimate the boundary of persistent activity. This boundary is defined as the curve which separates networks without persistent activity (above the curve) from networks with persistent activity (below the curve). To estimate the boundary of persistent activity, for a fixed percentage of impairment, we start from the highest level of impairment and observe the dynamics of the network. If the network activity is not persistent, then the level of impairment is decreased until a level of impairment with persistent activity is found (or the level of impairment reaches zero). Thus, for a fixed percentage of impairment, the boundary of persistent activity shows the maximum level of impairment that allows the network to have persistent activity. After the maximum level of impairment for a fixed percentage of impairment is found, the percentage of impairment is increased, and the networks are re-examined for persistent activity to find the maximum level of impairment for the new fixed percentage of impairment. This process continues until the percentage of impairment is 100%. The percentage of activity and the level of impairment are varied in increments of 10% and 0.1, respectively, to find the boundary of persistent activity.

The quality of the network activity is the second dynamical metric we introduce. We define it as the fraction of all neurons that fire at least once during a time window at the end of a longer period. Thus, this metric is useful for networks at the limit of persistent activity. Higher quality of activity means that more neurons participate in the activity of the network, during the time window used.

### Simulations

All the neurons in the network are initially at rest. At time *t* = 0, they receive an external current with a random uniform distribution between 0 and 1 μA/cm^2^. At *t* = 100 ms, the external current is removed and the dynamics of the network is observed until *t* = 4000 ms. All results presented are average values obtained from 50 realizations of the network with the same degree distribution. Error bars represent standard deviation of the measured values for these realizations. All results are obtained using custom MATLAB codes and the numerical integration of the network is performed using the ODE45 function.

## Results

**Figure 3:**
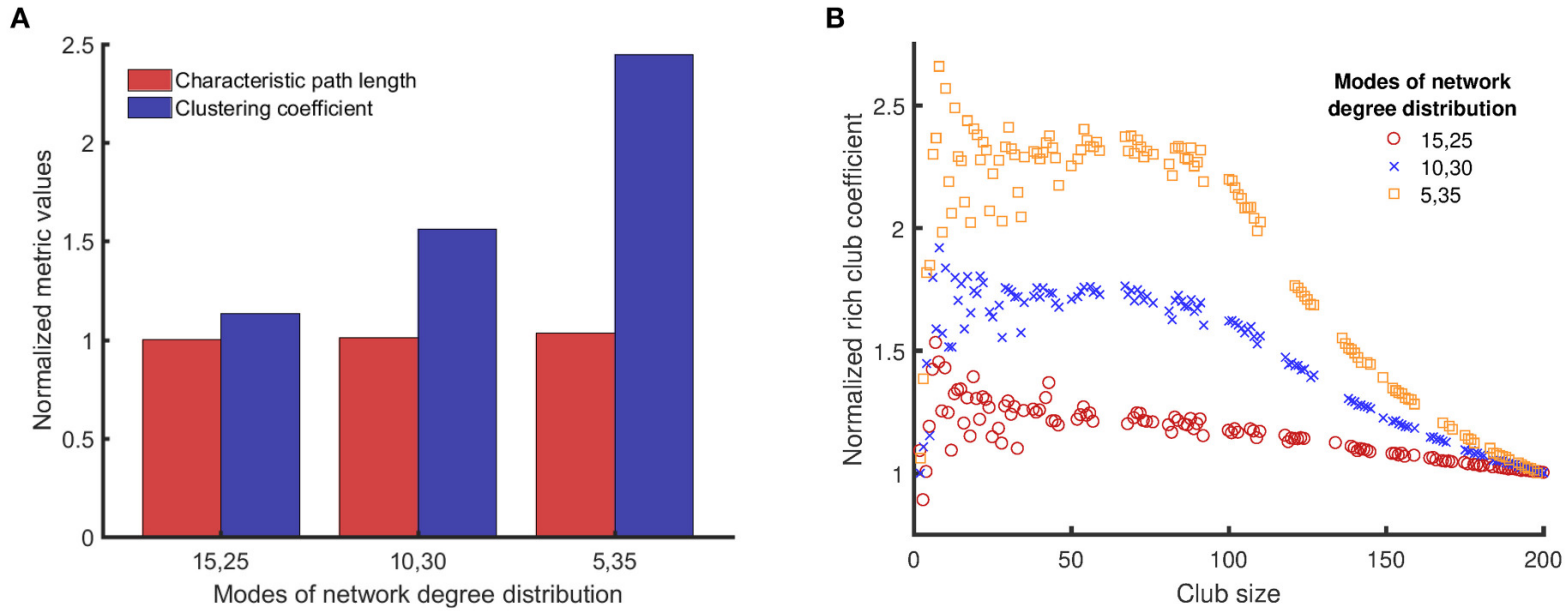
Comparison of clustering coefficients, characteristic path lengths, and rich club coefficients (the three topological metrics introduced in Section Topological Metrics) for three different network structures/topologies, each one with two modes of equal weight and mean degree values in pairs of {15, 25}, {10, 30}, and {5, 35}. The networks with distinct modes can resemble the structure of small world networks (high clustering coefficient) **(A)**, and can have rich clubs at the same time (rich club coefficient) **(B)**. The average of the two numbers is the same (20), and a reference network of {20, 20} represents a single modal distribution with a mean of 20. The topological metrics for random networks (single mean degree value of 20) are used to normalize the same topological metrics for networks with bimodal degree distribution to avoid artifacts in results related to the size of the networks and the probability of connectivity. For example, if we denote clustering coefficient by C, normalized clustering coefficient of networks with {15, 25} mean degree values is *C*_*normalized*_ = *C*_{15, 25}_ /*C*_{20}_.

First, we investigated how the topological metrics vary among the different network structures we have studied in this work. The topological metrics shown in Figure 3 explain why the dynamical metrics of different network structures vary.

Figure 3A shows that the normalized characteristic path lengths of different networks are near 1, which means that all these networks have characteristic path lengths similar to a purely random network. However, the normalized clustering coefficients of these networks are larger than 1, and the value of this metric increases with the difference between the mean values of the bimodal distribution. Figure 3B shows how the normalized rich club coefficients for different networks change with the club size. The normalized rich club coefficient approaches 1 when the club size reaches the size of the original network, as expected for this metric. All networks with bimodal degree distribution have normalized rich club coefficients >1 for many club sizes, which means that the nodes with larger degrees are connected to each other more in these networks compared to random networks. Moreover, for club sizes smaller than 100, the normalized rich club coefficients for networks with bimodal degree distributions is large and remains constant with small fluctuations. The value of this plateau region becomes higher as the distance between the modes of the degree distribution increases.

**Figure 4:**
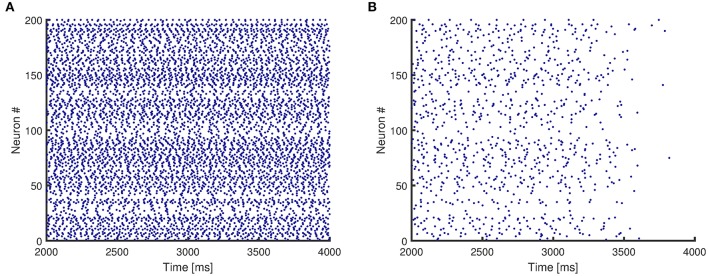
Raster plots for activity of a network with mean degree values of {10, 30}. **(A)** The original network, without any impairment, shows persistent activity. **(B)** The synapses of the original network are impaired randomly, and the network no longer shows persistent activity.

Figure 4 presents the raster plots for a sample case of network activity. Figure 4A shows that the original network without any impairment continues its activity after the initial stimulus is removed, and therefore even though it consists of only excitatory neurons, it reaches a stable state. In addition, Figure 4B shows that the same network structure loses persistent activity when its synapses are randomly impaired.

**Figure 5:**
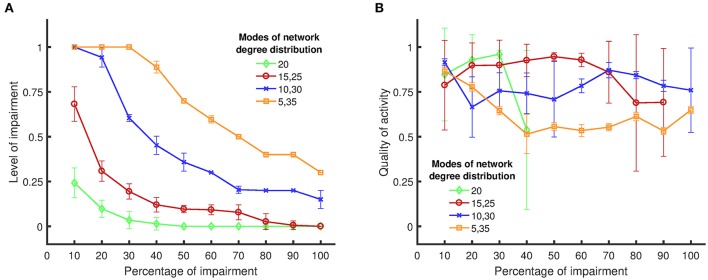
Boundary of persistent activity and quality of activity for four different network structures/topologies, when targets of impairments are chosen randomly. **(A)** The level of impairment (namely, the strength of an impaired synapse compared to a healthy synapse) implemented in the network, in relation to the applied percentage of impairment (namely, the percentage of synapses in the network that are weakened by the implemented level of impairment). The results show that random networks exhibit more vulnerability to synaptic loss compared to nonrandom networks. **(B)** The quality of activity (namely, the fraction of active neurons in the network), in relation to the percentage of applied impairment. Details for the definitions and the metrics can be found in Sections Impairment Modeling and Dynamical Metrics. Standard deviations from 50 separate realizations are shown by the error bars.

Next, we investigated how the network structure influences the dynamical metrics of the network by using four different degree distributions. The first degree distribution has only one mode with mean degree value of 20, which resembles a purely random network. The remaining three distributions have two modes with equal weights and have mean degree values in pairs of {15, 25}, {10, 30}, and {5, 35}. Figure 5 shows the boundary of persistent activity and the quality of activity when these networks are subjected to random impairments.

For each degree distribution, networks below the boundary have persistent activity (similar to Figure 4A), and networks above the boundary have lost their persistent activity (similar to Figure 4B). Figure 5A clearly shows that the purely random network contains the smallest region in which the persistent activity is maintained when impairments are imposed. These results suggest that random networks are most vulnerable to random impairments, and they cannot withstand any level of impairment when more than 40% of the network is damaged. In contrast, networks with bimodal degree distributions endure impairments considerably better than random networks, as their boundary of persistent activity is well above the boundary of persistent activity for random networks.

When the difference between the two mean values of the degree distribution increases, the neurons start to form two clusters with one cluster having higher rich club coefficient than the other (Figure 3B). The quality of activity can be used to determine whether neurons of only one of these clusters participate in the persistent activity of the networks. Since the weights of the two modes are equal, the number of neurons in each cluster is the same. Thus, if the quality of activity is above 0.5, then we can conclude that more than half the neurons are active (in the time window where the persistent activity was determined). Figure 5B shows the quality of activity for each of the networks at their own boundary of persistent activity. Note that not all networks show persistent activity at all impairment levels up to 100%. Hence, the plots stop at lower values of impairment because the quality of activity is not defined for higher levels of impairment. For example, the quality of activity cannot be defined for impairments over 40% for networks with one mode of degree distribution (and mean degree value of 20) as shown in Figure 5B. Similarly, the quality of activity cannot be defined for {15, 25} networks for impairments over 90%. Note, however, that the quality of activity is over 0.5 for all cases, which shows that all networks have more than one active cluster. However, the networks with {5, 35} modes, which have the largest region of persistent activity, have the poorest quality of activity compared to the rest of the networks. Therefore, the graphs in Figure 5 suggest that higher resistance to impairments has the downside of reducing the number of neurons that participate in the activity of the whole network. For instance, {15, 25} networks have comparable quality of activity to {10, 30} and {5, 35} networks even though their self-sustained activity region is smaller.

**Figure 6:**
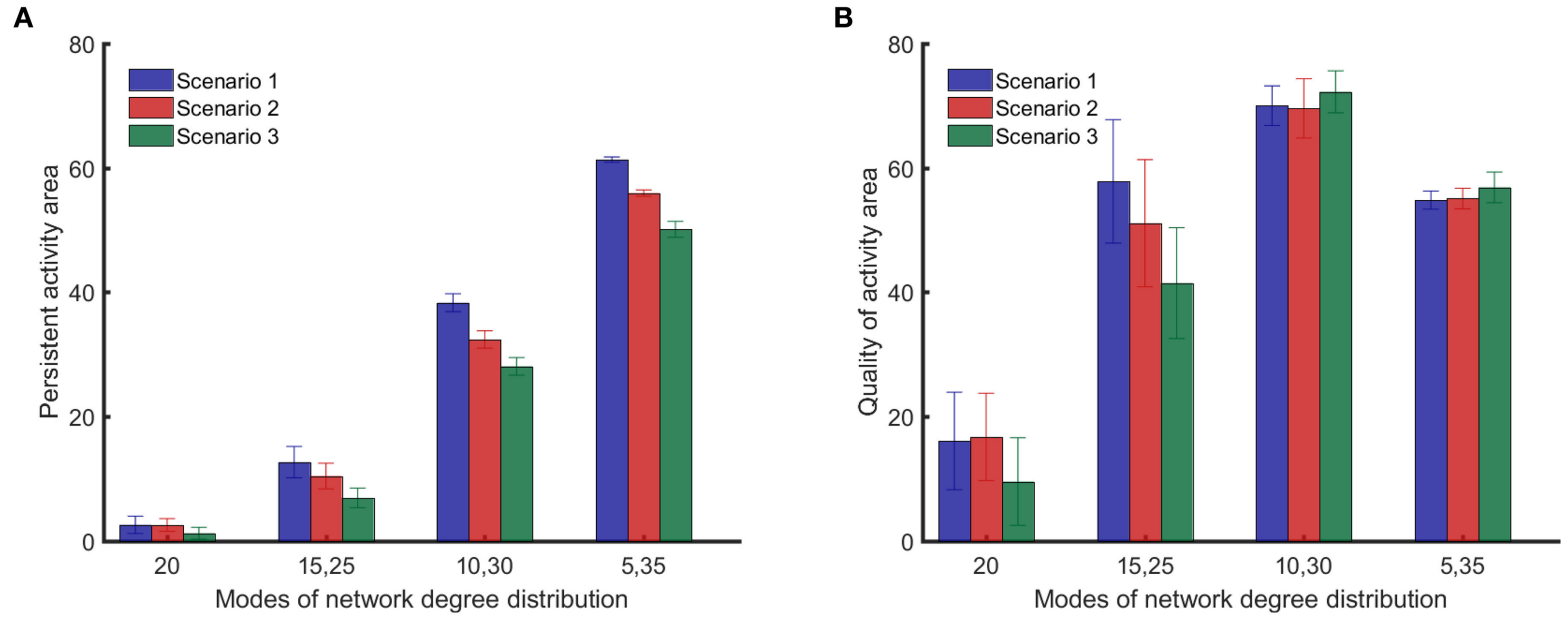
Comparison of persistent activity and quality of activity (the two dynamical metrics introduced in Section Dynamical Metrics), for different methods of impairment and network structures. In the first scenario, synapses are randomly impaired. In the second scenario, neurons with more synapses are preferably impaired. In the third scenario, synapses of most highly active neurons are preferably impaired. Details for the scenarios of impairment are provided in Section Impairment Modeling. **(A)** Persistent activity area suffers more from nonrandom impairment for all network structures. **(B)** Quality of activity area does not depend strongly on method of impairment for networks with distinct modes in their degree distribution. Standard deviations from 50 separate realizations are shown by the error bars.

Figure 6A shows that the integrated persistent activity corresponding to random impairments of synapses (scenario 1) is always higher than the integrated persistent activity for the other impairment scenarios for all network structures/topologies. Moreover, the integrated persistent activity corresponding to impairments of synapses of highly active neurons (scenario 3) is always lower than the integrated persistent activity for the other scenarios, which means that the persistent activity of neuronal networks suffers the most in this scenario. However, the comparison between the scenarios of impairment in Figure 6A does not hold for the quality of activity, as shown in Figure 6B, except for {15, 25} networks. Particularly, the quality of activity for {10, 30} and {5, 35} networks changes <4 between different impairment scenarios. All results in Figure 6 were analyzed in pairs by the unpaired two-tailed Student's *t*-test to determine if they are statistically significant (the performed *t*-test has 98 degrees of freedom for all the cases). For the results in Figure 6A, all the comparisons showed *p*-values smaller than 0.001, except for the comparison between scenarios 1 and 2 for random networks, which showed a *p*-value of 0.90. For the results in Figure 6B, all the comparisons showed *p*-values smaller than 0.001, except for the comparison between scenarios 1 and 2 for random networks (*p* = 0.63), {15, 25} networks (*p* = 0.002), {10, 30} networks (*p* = 0.057) and {5, 35} networks (*p* = 0.041).

**Figure 7:**
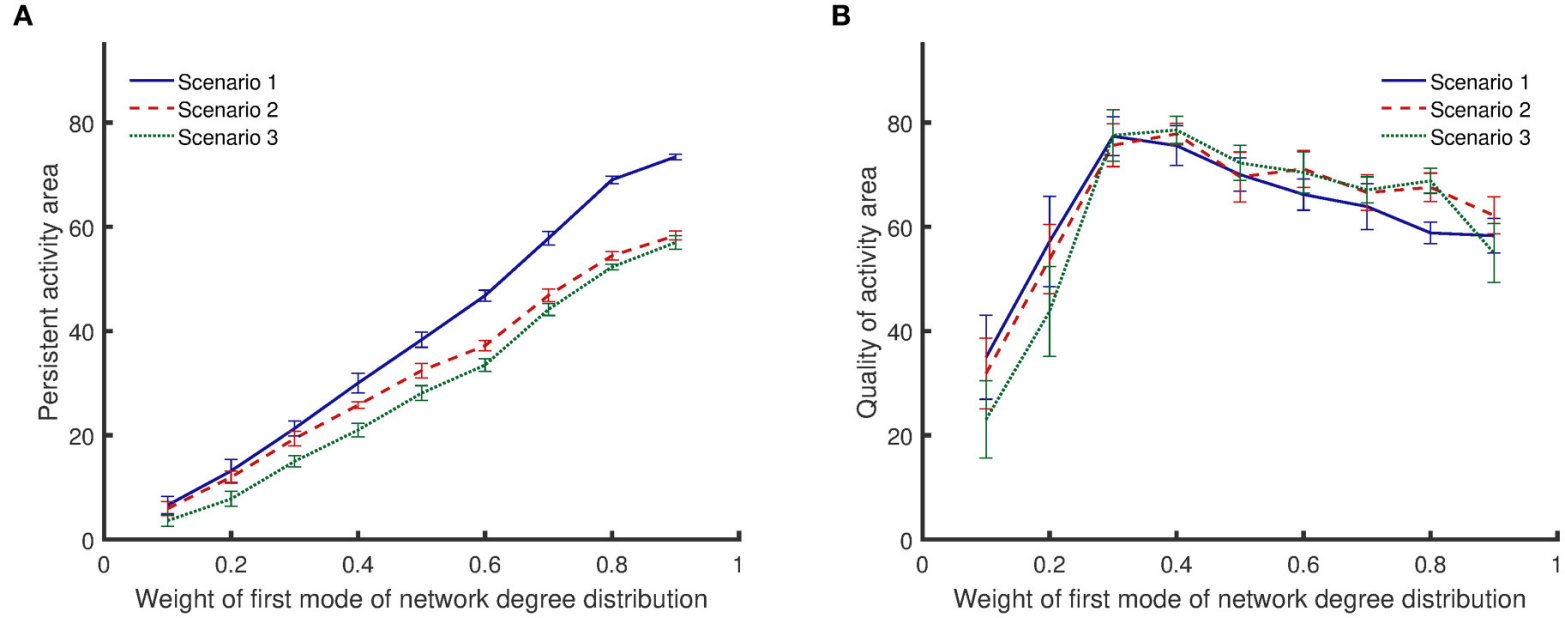
Comparison of persistent activity and quality of activity (the two dynamical metrics introduced in Section Dynamical Metrics), when the weights of degree distribution mode vary. In the first scenario, synapses are randomly impaired. In the second scenario, neurons with more synapses are preferably impaired. In the third scenario, synapses of most highly active neurons are preferably impaired. Details for the scenarios of impairment are provided in Section Impairment Modeling. **(A)** persistent activity area increases monotonically with weight of first mode of network degree distribution. **(B)** Quality of activity area initially increases with weight of first mode of network degree distribution, but it decreases with further increase of the weight. Standard deviations from 50 separate realizations are shown by the error bars.

All results presented above correspond to bimodal degree distributions with modes that have equal weights. However, different network structures can be constructed by keeping the mean value of one mode constant and varying the weights of each mode (sum of the weights must equal to 1). As the weight of the first mode becomes larger, the mean value of the second mode starts to increase to keep the total mean value constant. The results in Figure 6 show that {10, 30} networks exhibit both a high level of persistent activity and a high quality of activity. Therefore, it is insightful to vary the weights and examine networks where one of the mean values is kept at 10 while the other mean value is fixed by the weights. Figure 7 shows how the weights affect the dynamical metrics of different networks. When the weights are equal to 0.5, the results are the same as {10, 30} networks.

Figure 7A shows that the area of persistent activity region increases monotonically when the weight of the first mode increases. Similar to the results shown in Figure 6A, impairment scenario 1 inflicts less damage to the persistent activity compared to the other two scenarios. In addition, scenario 3 has the most invasive effect. For low weights of the first mode, the integrated persistent activity for all impairment scenarios is similar—the lines corresponding to scenarios 1 and 2 are close to each other. However, they start to separate as the weight of the first mode increases. In contrast, the integrated persistent activity for scenarios 2 and 3 are separated for low weight of the first mode, but the distance between them decreases slightly as the weight increases.

Figure 7B shows that the quality of activity changes nonlinearly with the weight of the first mode. Lines corresponding to different impairment scenarios cross each other several times. Therefore, unlike the persistent activity shown in Figure 7A, a general statement cannot be made about how different impairment scenarios affect the quality of activity. For low weights of the first mode, the quality of activity is low because the network has low integrated persistent activity. The quality of activity improves as the integrated persistent activity increases. However, further increasing the weight of the first mode lowers the quality of activity even though the integrated persistent activity is still improving.

## Discussion

Our results show the vulnerability of random networks to synaptic loss, compared to networks with bimodal degree distribution. The robustness of networks with bimodal degree distribution can be attributed to their topological metrics, and especially the presence of rich clubs. Our results also show that targeted synaptic loss, which may resemble different pathological or biological conditions, affects the dynamics of networks more, compared to random impairments. Therefore, monitoring the activity of networks has the potential to reveal underlying pathological or biological conditions earlier than symptom-based detection methods.

We have used a model based on the Hodgkin-Huxley formalism that has been previously used successfully to simulate dynamics of neuronal networks (Fink et al., 2011). One advantage of this model is its capability to be switched to a type 2 neuron with the addition of a slow potassium current, which is ultimately responsible for the shift in neural excitability mediated by ACh (Fink et al., 2011). Ca^2+^ dynamics have been shown to be related with persistent activity of neurons (Fransén et al., 2006; Neymotin et al., 2016). However, even though Ca^2+^ dynamics are not captured in this model, previous research has shown that persistent activity can be observed on the network level even when simple integrate-and-fire neurons have been used (Roxin et al., 2004). Like many other network simulations, details of simulations such as the values of dynamical metrics and boundary of persistent activity will change, because the elements of the network (neurons) will change. However, our main goal is to examine whether the network topology and pattern (or scenario) of impairment has significant counter-intuitive (or non-intuitive) effects on the network function. Hence, it is the relative robustness that we are mostly interested in; relative between different impairment scenarios or network topologies. Therefore, we believe that using other models such as the ones that include Ca^2+^ dynamics will not change the conclusions of this work about the influence of network structure on the activity of the network.

As a first step, we used the degree distribution of networks to construct networks with different topological metrics. While completely random connectivity topologies are usually the first choice made when studying the dynamics of neuronal networks, *in vitro* (Downes et al., 2012; Schroeter et al., 2015), *in vivo* (Ball et al., 2014) and even *in silico* (Izhikevich and Edelman, 2008) studies have revealed that neurons form structures/topologies which are correlated to their functionality. One simple way to explain the structure/topology of a network is through its degree distribution (Newman, 2010). We built different network structures/topologies by combining Poisson distributions (the degree distribution of a random network) with two different mean values. This method of network generation creates networks with bimodal degree distributions, which consist of rich clubs with high clustering coefficients, while their characteristic path lengths are almost equal to that of random networks. Networks with high clustering coefficients and path lengths comparable to random networks are known to have features of small world networks (Watts and Strogatz, 1998). The characteristic path lengths of all the networks we studied are close to the characteristic path lengths of random networks. Therefore, networks with higher clustering coefficients are more similar to small world networks. Our results show that the clustering coefficient increases as the distance between modes of degree distribution increases (Figure 3). At the same time, the increased distance between modes of degree distribution leads to an increase in the persistent activity area. Hence, our results show that networks that have properties of small world networks are more robust. We define robustness as the ability of neuronal networks to maintain their persistent activity when exposed to impairments. Small world networks can tolerate impairments better than random networks since the connections between neurons in small world networks have more closed loops to sustain the activity of the whole network. Other studies (Roxin et al., 2004; Shanahan, 2008), have also reported that small world networks are more likely to have persistent activity. However, those reports are not parallel to our work since they do not consider impairment in synaptic connections. Moreover, their networks are built following the conventional method of constructing small world networks, so they do not consist of distinct rich clubs. However, features of small world networks are likely not enough to explain the higher robustness of networks with bimodal degree distribution, because this topological metric does not describe the variation in the quality of activity for different networks.

The rich club coefficients can be used to describe both the persistent activity and the quality of activity for networks with bimodal degree distribution. When networks start to form rich clubs, hubs of highly connected neurons are created, which are also interconnected to each other. During impairments, these hubs can preserve the activity of the whole network. Having a core of highly connected neurons enables such network structures/topologies to maintain self-sustained activity when they experience loss of synapses. Moreover, for such networks, removal of connections between members of rich clubs and neurons outside the rich clubs does not influence the persistent activity significantly because neurons outside the rich clubs are not responsible for maintaining the persistent activity. Neurons in the rich club are also connected to the neurons outside the rich clubs. Hence, they distribute the activity to the whole network. This is the reason why high rich club coefficients coincide with high robustness in our results (Figures 3B, 6A).

Our results suggest that there is a compromise between quality of activity and persistent activity of neuronal networks. Unlike the integrated persistent activity, the integrated quality of activity shows a nonlinear behavior when the distance between the modes of the degree distribution increases (Figure 6B). Such behavior is more obvious when the weights of modes are changed (Figure 7B). Initially, the quality of activity is low when the distance between the modes of degree distribution is small, because the integrated persistent activity is low. Such networks resemble random networks rather than networks with bimodal degree distributions. When the distance between the modes of degree distribution increases, the integrated quality of activity and the integrated persistent activity both increase. In these networks, rich clubs sustain the activity of the whole network and since they are also connected to neurons outside rich clubs, they are able to keep neurons outside rich clubs active as well. However, as the distance between the modes of degree distribution increases, the robustness of networks continues to increase, whereas the quality of activity starts to decrease. At the same time, neurons outside rich clubs make few connections, either with each other or with members of rich clubs. In fact, the sharp transition in the normalized rich club coefficients of {5, 35} networks (Figure 3B) indicates that the core has weak connection with neurons outside the core. Therefore, neurons outside of rich clubs lose their activity even when a few of their synapses are removed. In this situation, rich clubs fail to act as a driving force for the rest of the network. Hence, the quality of activity for such networks is low even though highly connected hubs that form rich clubs can maintain the activity of the whole network.

The interplay between persistent activity and quality of activity can be considered as an optimization problem. To achieve higher robustness, our results suggest that the number of connections between neurons in rich clubs must increase. However, if the size of the neuronal network and its synapses are constrained to remain the same, then more connections between neurons in rich clubs mean fewer connections between neurons outside rich clubs. Therefore, even though such networks can endure impairments very well and can maintain persistent activity, only few neurons participate in the activity of the whole network and the quality of activity remains low. From this perspective, the network structure/topology can be viewed as a multi-objective optimization problem where the fitness of a network can be determined by both persistent and quality of activity, and the number of neurons and synapses are the constraints. Even though the network optimization can be regarded as an abstract mathematical problem, emergence of certain structures/topologies in networks can also be considered as evolution of these networks in reality (Holland, 1992). However, a random network can evolve into different network structures/topologies to serve different tasks (Hiratani and Fukai, 2016). For example, Sporns et al. (2000) have shown that based on differently imposed criteria, their graph selection algorithm leads to networks with different structures and capabilities. Although we have not solved such an optimization problem in the present work, our results show that networks with bimodal distributions have good fitness. Therefore, if a neuronal network requires high robustness to perform its tasks, a network with bimodal degree distribution can be the plausible solution. More precisely, networks with bimodal degree distributions with a moderate distance between distinct modes have high robustness and high quality of activity at the same time. Moreover, our results suggest that random networks are the least preferable neuronal network structure/topology for the metrics we have used, since such networks have neither high persistent activity nor high quality of activity.

In the present study, we have explored also how selective impairment of neurons can affect the dynamics of neuronal networks by investigating targeted weakening of synapses. We have explored how three different scenarios of synapses loss can affect the dynamical features of neuronal networks.

In the first impairment scenario, synapses are impaired randomly, leading to the least impact on the persistent activity for all the network structures/topologies (Figures 6A, 7A). The importance of all neurons and their synapses in the activity of the whole network is not the same, especially for the neuronal networks that do not have random connectivity. However, the reason why random impairments are the least damaging method is not the fact that synapses of more important neurons are not selected. Essentially, the likelihood of damaging such neurons is the same as any other neuron since the method of impairment is random. In fact, highly damaging effects of impairing critical neurons are compensated by impairing synapses of neurons that are less important to the activity of the whole network. Therefore, random impairments lead to overall less damaging effects compared to other impairment scenarios we have described in our study.

In the second impairment scenario, synapses of neurons with larger number of synapses are more likely to be impaired, leading to more damaging effects to the persistent activity of neuronal networks than random impairments of neurons, for all network structures/topologies. The number of synapses a neuron has is a topological feature of a neuronal network. Therefore, if the wiring between neurons in a network is known, this wiring can be used to suggest where the impairments will occur in case of damaged axonal transport. van den Heuvel and Sporns (2011) have performed a similar analysis by observing the efficacy of neuronal networks. They have shown that targeted impairments that remove the links between members of rich clubs in the network induce a more dramatic change on the efficacy of neuronal networks than random impairments. The mechanism used for the targeted impairments in that study is not mentioned, but such impairments resemble our second scenario. Complimentary to our speculation about the links between the hub location and axonal transport deficiencies, experimental results have also shown that the hub locations correlate with Aβ deposition in Alzheimer's disease (Buckner et al., 2009). Hence, hub locations can be monitored to detect abnormalities in neuronal networks earlier and with more efficiency.

In the third impairment scenario, synapses of highly active neurons are more likely to be impaired, leading to the most destructive effect on the persistent activity of all neuronal networks, when compared to the other impairment scenarios. The structure/topology of neuronal networks plays an important role, but the dynamics is important also. The dynamic map of activity in neuronal networks can provide critical information about regions of interest. Other research has similarly suggested that regions of high activity and metabolism can be associated with cellular mechanism involved in Alzheimer's disease (Buckner et al., 2009). Moreover, it has been proposed that, highly active neurons in the brain can be especially vulnerable to intrinsic oxidative stress, thus being susceptible to functional decline during normal aging or neurodegenerative diseases (Wang and Michaelis, 2010). Therefore, monitoring the activity of neuronal networks can reveal the critical regions and neurons that influence the most the activity of the whole network. Consequently, we speculate that losses or changes in the activity of such regions can be used as an early sign of deficiencies in neuronal networks.

Altogether, we speculate that the transition in the network structure can be used as an indicator of neurodegenerative disease, since the robustness of neuronal networks decreases when they lose their structured topology. Such transition of the brain network toward randomness has already been shown even in normal aging (Knyazev et al., 2015). Therefore, monitoring alterations in the brain network structure has the potential to be used as an early diagnostic method in neurodegenerative diseases. Moreover, our results show that even though the topological metrics and maps of neuronal networks can provide valuable information, they should be accompanied by the dynamical metrics and maps of neuronal networks that are even more informative. Our results illustrate that such an argument is even stronger when neuronal networks are not randomly connected, and are instead topologically defined.

## Author contributions

EM and BE conceived the idea. EM, EG, VB, and BE designed the experiments. EM created the computational model and performed the simulations. EM, EG, VB, and BE analyzed the data. EM and BE contributed analysis tools. EM and EG wrote the manuscript. All authors reviewed, revised and approved the final manuscript.

### Conflict of interest statement

The authors declare that the research was conducted in the absence of any commercial or financial relationships that could be construed as a potential conflict of interest.

## References

[B1] Abbott L. F. Nelson S. B. (2000). Synaptic plasticity: taming the beast. Nat. Neurosci. 3, 1178–1183. 10.1038/81453 11127835

[B2] Abuhassan K. Coyle D. Maguire L. (2014). Compensating for thalamocortical synaptic loss in Alzheimer's disease. Front. Comput. Neurosci. 8:65. 10.3389/fncom.2014.00065 24987349 PMC4060454

[B3] Achard S. (2006). A resilient, low-frequency, small-world human brain functional network with highly connected association cortical hubs. J. Neurosci. 26, 63–72. 10.1523/JNEUROSCI.3874-05.2006 16399673 PMC6674299

[B4] Amitai Y. (1994). Membrane potential oscillations underlying firing patterns in neocortical neurons. Neuroscience 63, 151–161. 10.1016/0306-4522(94)90013-2 7898645

[B5] Baddeley A. (1992). Working memory. Science 255, 556–559. 10.1126/science.1736359 1736359

[B6] Baddeley A. D. Baddeley H. A. Bucks R. S. Wilcock G. K. (2001). Attentional control in Alzheimer's disease. Brain 124, 1492–1508. 10.1093/brain/124.8.1492 11459742

[B7] Baddeley A. D. Bressi S. Della Sala S. Logie R. Spinnler H. (1991). The decline of working memory in Alzheimer's disease. A longitudinal study. Brain 114(Pt 6), 2521–2542. 10.1093/brain/114.6.2521 1782529

[B8] Ball G. Aljabar P. Zebari S. Tusor N. Arichi T. Merchant N. (2014). Rich-club organization of the newborn human brain. Proc. Natl. Acad. Sci. U.S.A. 11, 7456–7461. 10.1073/pnas.1324118111 PMC4034228 24799693

[B9] Barrat A. Barthelemy M. Vespignani A. (2008). Dynamical Processes on Complex Networks. Cambridge: Cambridge University Press

[B10] Buckner R. L. Sepulcre J. Talukdar T. Krienen F. M. Liu H. Hedden T.. (2009). Cortical hubs revealed by intrinsic functional connectivity: mapping, assessment of stability, and relation to Alzheimer's Disease. J. Neurosci. 29, 1860–1873. 10.1523/JNEUROSCI.5062-08.2009 19211893 PMC2750039

[B11] Cohen R. Havlin S. (2010). Complex Networks. Cambridge: Cambridge University Press.

[B12] Colizza V. Flammini A. Serrano M. A. Vespignani A. (2006). Detecting rich-club ordering in complex networks. Nat. Phys. 2, 110–115. 10.1038/nphys209

[B13] Crossley N. A. Mechelli A. Scott J. Carletti F. Fox P. T. McGuire P.. (2014). The hubs of the human connectome are generally implicated in the anatomy of brain disorders. Brain 137, 2382–2395. 10.1093/brain/awu132 25057133 PMC4107735

[B14] Curtis C. E. D'Esposito M. (2003). Persistent activity in the prefrontal cortex during working memory. Trends Cogn. Sci. 7, 415–423. 10.1016/S1364-6613(03)00197-9 12963473

[B15] de Reus M. A. van den Heuvel M. P. (2013). Rich club organization and intermodule communication in the cat connectome. J. Neurosci. 33, 12929–12939. 10.1523/JNEUROSCI.1448-13.2013 23926249 PMC6619725

[B16] Destexhe A. Mainen Z. F. Sejnowski T. J. (1998). Kinetic Models of Synaptic Transmission: From Ions to Networks. Methods Neural Model from Ions to Networks. Cambridge, MA: MIT Press.

[B17] De Vos K. J. Grierson A. J. Ackerley S. Miller C. C. J. (2008). Role of axonal transport in neurodegenerative diseases. Annu. Rev. Neurosci. 31, 151–173. 10.1146/annurev.neuro.31.061307.090711 18558852

[B18] Downes J. H. Hammond M. W. Xydas D. Spencer M. C. Becerra V. M. Warwick K.. (2012). Emergence of a small-world functional network in cultured neurons. PLoS Comput. Biol. 8:e1002522. 10.1371/journal.pcbi.1002522 22615555 PMC3355061

[B19] Eguíluz V. M. Chialvo D. R. Cecchi G. A. Baliki M. Apkarian A. V. (2005). Scale-free brain functional networks. Phys. Rev. Lett. 94:18102. 10.1103/PhysRevLett.94.018102 15698136

[B20] Erdös P. Rényi A. (1959). On random graphs. Publ. Math. 6, 290–297.

[B21] Fink C. G. Booth V. Zochowski M. (2011). Cellularly-driven differences in network synchronization propensity are differentially modulated by firing frequency. PLoS Comput. Biol. 7:e1002062. 10.1371/journal.pcbi.1002062 21625571 PMC3098201

[B22] Fornito A. Zalesky A. Breakspear M. (2013). Graph analysis of the human connectome: promise, progress, and pitfalls. Neuroimage 80, 426–444. 10.1016/j.neuroimage.2013.04.087 23643999

[B23] Fransén E. Tahvildari B. Egorov A. V. Hasselmo M. E. Alonso A. A. Stern C. E. (2006). Mechanism of graded persistent cellular activity of entorhinal cortex layer v neurons. Neuron 49, 735–746. 10.1016/j.neuron.2006.01.036 16504948

[B24] Gaál Z. A. Boha R. Stam C. J. Molnár M. (2010). Age-dependent features of EEG-reactivity—Spectral, complexity, and network characteristics. 479, 79–84. 10.1016/j.neulet.2010.05.037 20560166

[B25] Hass J. Hertäg L. Durstewitz D. (2016). A detailed data-driven network model of prefrontal cortex reproduces key features of *in vivo* activity. PLoS Comput. Biol. 12:e1004930. 10.1371/journal.pcbi.1004930 27203563 PMC4874603

[B26] He Y. Chen Z. J. Evans A. C. (2007). Small-world anatomical networks in the human brain revealed by cortical thickness from MRI. Cereb. Cortex 17, 2407–2419. 10.1093/cercor/bhl149 17204824

[B27] Hiratani N. Fukai T. (2016). Hebbian wiring plasticity generates efficient network structures for robust inference with synaptic weight plasticity. Front. Neural Circuits 10:41. 10.3389/fncir.2016.00041 27303271 PMC4885844

[B28] Hodge M. R. Horton W. Brown T. Herrick R. Olsen T. Hileman M. E.. (2016). ConnectomeDB—Sharing human brain connectivity data. Neuroimage 124, 1102–1107. 10.1016/j.neuroimage.2015.04.046 25934470 PMC4626437

[B29] Holland J. H. (1992). Adaptation in Natural and Artificial Systems: an Introductory Analysis with Applications to Biology, Control, and Artificial Intelligence. Cambridge, MA: Bradford Books, MIT Press.

[B30] Izhikevich E. M. Edelman G. M. (2008). Large-scale model of mammalian thalamocortical systems. Proc. Natl. Acad. Sci. U.S.A. 105, 3593–3598. 10.1073/pnas.0712231105 18292226 PMC2265160

[B31] Kamat P. K. Kalani A. Rai S. Swarnkar S. Tota S. Nath C.. (2016). Mechanism of oxidative stress and synapse dysfunction in the pathogenesis of Alzheimer's Disease: understanding the therapeutics strategies. Mol. Neurobiol. 53, 648–661. 10.1007/s12035-014-9053-6 25511446 PMC4470891

[B32] Knyazev G. G. Volf N. V. Belousova L. V. (2015). Age-related differences in electroencephalogram connectivity and network topology. Neurobiol. Aging 36, 1849–1859. 10.1016/j.neurobiolaging.2015.02.007 25766772

[B33] Kocher M. Gleichgerrcht E. Nesland T. Rorden C. Fridriksson J. Spampinato M. V.. (2015). Individual variability in the anatomical distribution of nodes participating in rich club structural networks. Front. Neural Circuits 9:16. 10.3389/fncir.2015.00016 25954161 PMC4405623

[B34] Kopeikina K. J. Wegmann S. Pitstick R. Carlson G. A. Bacskai B. J. Betensky R. A.. (2013). Tau causes synapse loss without disrupting calcium homeostasis in the rTg4510 model of tauopathy. PLoS ONE 8:e80834. 10.1371/journal.pone.0080834 24278327 PMC3835324

[B35] Kosik K. S. (2013). Diseases: study neuron networks to tackle Alzheimer's. Nature 503, 31–32. 10.1038/503031a 24218661

[B36] Marder E. Thirumalai V. (2002). Cellular, synaptic and network effects of neuromodulation. Neural Networks 15, 479–493. 10.1016/S0893-6080(02)00043-6 12371506

[B37] McAuley J. J. da Fontoura Costa L. Caetano T. S. (2007). Rich-club phenomenon across complex network hierarchies. Appl. Phys. Lett. 91, 84103 10.1063/1.2773951

[B38] Morgan R. J. Soltesz I. (2008). Nonrandom connectivity of the epileptic dentate gyrus predicts a major role for neuronal hubs in seizures. Proc. Natl. Acad. Sci. U.S.A. 105, 6179–6184. 10.1073/pnas.0801372105 18375756 PMC2299224

[B39] Newman M. (2010). Networks. Oxford: Oxford University Press.

[B40] Neymotin S. A. McDougal R. A. Bulanova A. S. Zeki M. Lakatos P. Terman D.. (2016). Calcium regulation of HCN channels supports persistent activity in a multiscale model of neocortex. Neuroscience 316, 344–366. 10.1016/j.neuroscience.2015.12.043 26746357 PMC4724569

[B41] Oh S. W. Harris J. A. Ng L. Winslow B. Cain N. Mihalas S.. (2014). A mesoscale connectome of the mouse brain. Nature 508, 207–214. 10.1038/nature13186 24695228 PMC5102064

[B42] Palop J. J. Chin J. Mucke L. (2006). A network dysfunction perspective on neurodegenerative diseases. Nature 443, 768–773. 10.1038/nature05289 17051202

[B43] Petersen P. C. Vestergaard M. Jensen K. H. R. Berg R. W. (2014). Premotor spinal network with balanced excitation and inhibition during motor patterns has high resilience to structural division. J. Neurosci. 34, 2774–2784. 10.1523/JNEUROSCI.3349-13.2014 24553920 PMC6608521

[B44] Pozzo-Miller L. D. Gottschalk W. Zhang L. McDermott K. Du J. Gopalakrishnan R.. (1999). Impairments in high-frequency transmission, synaptic vesicle docking, and synaptic protein distribution in the hippocampus of BDNF knockout mice. J. Neurosci. 19, 4972–4983. 10366630 10.1523/JNEUROSCI.19-12-04972.1999 PMC6782660

[B45] Rich S. Booth V. Zochowski M. (2016). Intrinsic cellular properties and connectivity density determine variable clustering patterns in randomly connected inhibitory neural networks. Front. Neural Circuits 10:82. 10.3389/fncir.2016.00082 27812323 PMC5071331

[B46] Rizzoli S. O. (2014). Synaptic vesicle recycling: steps and principles. EMBO J. 33, 788–822. 10.1002/embj.201386357 24596248 PMC4194108

[B47] Roxin A. (2011). The role of degree distribution in shaping the dynamics in networks of sparsely connected spiking neurons. Front. Comput. Neurosci. 5:8. 10.3389/fncom.2011.00008 21556129 PMC3058136

[B48] Roxin A. Riecke H. Solla S. A. (2004). Self-sustained activity in a small-world network of excitable neurons. Phys. Rev. Lett. 92:198101. 10.1103/PhysRevLett.92.198101 15169447

[B49] Rubinov M. Sporns O. (2010). Complex network measures of brain connectivity: uses and interpretations. Neuroimage 52, 1059–1069. 10.1016/j.neuroimage.2009.10.003 19819337

[B50] Sakai K. Rowe J. B. Passingham R. E. (2002). Active maintenance in prefrontal area 46 creates distractor-resistant memory. Nat. Neurosci. 5, 479–484. 10.1038/nn846 11953754

[B51] Schroeter M. S. Charlesworth P. Kitzbichler M. G. Paulsen O. Bullmore E. T. (2015). Emergence of rich-club topology and coordinated dynamics in development of hippocampal functional networks *in vitro*. J. Neurosci. 35, 5459–5470. 10.1523/JNEUROSCI.4259-14.2015 25855164 PMC4388914

[B52] Selkoe D. J. (2002). Alzheimer's Disease is a synaptic failure. Science 298, 789–791. 10.1126/science.1074069 12399581

[B53] Shanahan M. (2008). Dynamical complexity in small-world networks of spiking neurons. Phys. Rev. E 78:41924. 10.1103/PhysRevE.78.041924 18999472

[B54] Shankar G. M. Walsh D. M. (2009). Alzheimer's disease: synaptic dysfunction and Aβ. Mol. Neurodegener 4:48. 10.1186/1750-1326-4-48 19930651 PMC2788538

[B55] Sheng M. Sabatini B. L. Sudhof T. C. (2012). Synapses and Alzheimer's Disease. Cold Spring Harb. Perspect. Biol. 4, a005777–a005777. 10.1101/cshperspect.a005777 22491782 PMC3331702

[B56] Smit D. J. A. Boersma M. van Beijsterveldt C. E. M. Posthuma D. Boomsma D. I. Stam C. J.. (2010). Endophenotypes in a dynamically connected brain. Behav. Genet. 40, 167–177. 10.1007/s10519-009-9330-8 20111993 PMC2829652

[B57] Sporns O. (2010). Networks of the Brain. Cambridge, MA: MIT Press.

[B58] Sporns O. Tononi G. Edelman G. M. (2000). Theoretical neuroanatomy: relating anatomical and functional connectivity in graphs and cortical connection matrices. Cereb. Cortex 10, 127–141. 10.1093/cercor/10.2.127 10667981

[B59] Stiefel K. M. Gutkin B. S. Sejnowski T. J. (2009). The effects of cholinergic neuromodulation on neuronal phase-response curves of modeled cortical neurons. J. Comput. Neurosci. 26, 289–301. 10.1007/s10827-008-0111-9 18784991 PMC2857973

[B60] Stopford C. L. Thompson J. C. Neary D. Richardson A. M. T. Snowden J. S. (2012). Working memory, attention, and executive function in Alzheimer's disease and frontotemporal dementia. Cortex 48, 429–446. 10.1016/j.cortex.2010.12.002 21237452

[B61] Towlson E. K. Vertes P. E. Ahnert S. E. Schafer W. R. Bullmore E. T. (2013). The rich club of the *C. elegans* neuronal connectome. J. Neurosci. 33, 6380–6387. 10.1523/JNEUROSCI.3784-12.2013 23575836 PMC4104292

[B62] van den Heuvel M. P. Sporns O. (2011). Rich-club organization of the human connectome. J. Neurosci. 31, 15775–15786. 10.1523/JNEUROSCI.3539-11.2011 22049421 PMC6623027

[B63] van den Heuvel M. P. Stam C. J. Boersma M. Hulshoff Pol H. E. (2008). Small-world and scale-free organization of voxel-based resting-state functional connectivity in the human brain. Neuroimage 43, 528–539. 10.1016/j.neuroimage.2008.08.010 18786642

[B64] Van Essen D. C. Smith S. M. Barch D. M. Behrens T. E. J. Yacoub E. Ugurbil K. (2013). The WU-Minn human connectome project: an overview. Neuroimage 80, 62–79. 10.1016/j.neuroimage.2013.05.041 23684880 PMC3724347

[B65] Varshney L. R. Chen B. L. Paniagua E. Hall D. H. Chklovskii D. B. (2011). Structural properties of the *Caenorhabditis elegans* neuronal network. PLoS Comput. Biol. 7:e1001066. 10.1371/journal.pcbi.1001066 21304930 PMC3033362

[B66] Wang L. Li Y. Metzak P. He Y. Woodward T. S. (2010). Age-related changes in topological patterns of large-scale brain functional networks during memory encoding and recognition. Neuroimage 50, 862–872. 10.1016/j.neuroimage.2010.01.044 20093190

[B67] Wang X. Michaelis E. K. (2010). Selective neuronal vulnerability to oxidative stress in the brain. Front. Aging Neurosci. 2:12. 10.3389/fnagi.2010.00012 20552050 PMC2874397

[B68] Watts D. J. Strogatz S. H. (1998). Collective dynamics of “small-world” networks. Nature 393, 440–442. 10.1038/30918 9623998

[B69] White J. G. Southgate E. Thomson J. N. Brenner S. (1986). The structure of the nervous system of the nematode *Caenorhabditis elegans*. Philos. Trans. R. Soc. B Biol. Sci. 314, 1–340. 10.1098/rstb.1986.0056 22462104

[B70] Wu K. Taki Y. Sato K. Qi H. Kawashima R. Fukuda H. (2013). A longitudinal study of structural brain network changes with normal aging. Front. Hum. Neurosci. 7:113. 10.3389/fnhum.2013.00113 23565087 PMC3615182

[B71] Zhou S. Mondragon R. J. (2004). The rich-club phenomenon in the internet topology. IEEE Commun. Lett. 8, 180–182. 10.1109/LCOMM.2004.823426

[B72] Zhu W. Wen W. He Y. Xia A. Anstey K. J. Sachdev P.. (2012). Changing topological patterns in normal aging using large-scale structural networks. Neurobiol. Aging 33, 899–913. 10.1016/j.neurobiolaging.2010.06.022 20724031

